# Identification of Variable Lymphocyte Receptors That Target the Human Blood–Brain Barrier

**DOI:** 10.3390/pharmaceutics17091179

**Published:** 2025-09-10

**Authors:** Moriah E. Katt, Elizabeth A. Waters, Benjamin D. Gastfriend, Brantley R. Herrin, Max D. Cooper, Eric V. Shusta

**Affiliations:** 1 Department of Chemical and Biological Engineering, University of Wisconsin-Madison, Madison, WI 53706, USAeshusta@wisc.edu (E.V.S.); 2 Department of Chemical and Biomedical Engineering, West Virginia University, Morgantown, WV 26506, USA; 3 Department of Neuroscience, West Virginia University, Morgantown, WV 26506, USA; 4 Department of Pathology and Laboratory Medicine, Emory University, Atlanta, GA 30322, USAmdcoope@emory.edu (M.D.C.); 5 Department of Neurological Surgery, University of Wisconsin-Madison, Madison, WI 53706, USA

**Keywords:** brain drug delivery, blood–brain barrier, variable lymphocyte receptor

## Abstract

**Background/Objectives**: Receptor-mediated transcytosis utilizing the native transporters at the blood–brain barrier (BBB) is a growing strategy for the delivery of therapeutics to the brain. One of the major challenges in identifying appropriate human transcytosis targets is that there is a species-specific transporter expression profile at the BBB, complicating translation of successful preclinical candidates into humans. In an effort to overcome this obstacle and identify proteins capable of binding human-relevant BBB ligands, we generated and screened a BBB-targeting library against human-induced pluripotent stem cell-derived brain microvascular endothelial-like cells (iPSC-derived BMEC-like cells). As targeting molecules, we used lamprey antibodies, known as variable lymphocyte receptors (VLRs), and generated a VLR library by immunizing lamprey with iPSC-derived BMEC-like cells, and inserting the resultant VLR repertoire into the yeast surface display system. **Methods**: The yeast displayed VLR library was then panned against human iPSC-derived BMEC-like cells and lead VLRs were validated using human in vitro models and mouse and human ex vivo brain tissue sections. **Results**: Finally, brain uptake for a set of VLRs was validated in mice. Of the 15 lead VLR candidates, 14 bound to human BBB antigens, and 10 bound to the murine BBB. Pharmacodynamic testing using the neuroactive peptide neurotensin indicated that the lead candidate, VLR2G, could cross the mouse BBB after intravenous injection and deliver sufficient neurotensin payload to generate a pharmacological response and lower systemic body temperature. **Conclusions**: Together, these results demonstrate the application of a novel screening technique capable of identifying a VLR with human relevance that can cross the BBB and deliver a payload.

## 1. Introduction

Delivery of therapeutics to the brain is difficult, with many therapeutics having limited efficacy due to their restricted ability to pass through the blood–brain barrier (BBB) [1]. The BBB has specialized permeability properties as a result of its interactions with other members of the neurovascular unit, such as astrocytes, pericytes, and neurons. As a result, the BBB maintains brain homeostasis, meets brain energy and nutrient demands, and essentially eliminates all extraneous traffic, including that of potential therapeutics [2]. Models of the BBB are continuing to improve and capture more and more of the complexity of the physiological environment, but have historically still fallen short of accurately predicting the success of potential therapeutics, further complicating the brain drug delivery problem [3]. Thus, there is a clear need for novel approaches for the identification of brain-targeting molecules in order to improve the transition of brain therapies from research discoveries to clinical applications.

There are many different approaches that researchers have been exploring to improve therapeutic delivery through the BBB [4,5]. These methods include techniques like mechanical BBB disruption using focused ultrasound, biochemical BBB disruption, intracranial injection, and receptor-mediated transcytosis (RMT) [6,7]. RMT-based delivery leverages the targeting and co-opting of endogenous BBB transporters to facilitate targeted delivery to the brain [8,9]. The RMT-targeting approach is non-invasive and compatible with most standard routes of administration. In addition, RMT occurs through vesicular transport, thus allowing for the transport of larger molecules and nanocarriers, compared to methods reliant on passive diffusion [10]. Identification and targeting of RMT receptors has long been a proposed means of increasing drug delivery to the brain [5]. Antibodies are often used to target the RMT receptors and can be used to ferry conjugated drug cargo across the BBB. The most commonly used BBB RMT receptors are the transferrin receptor, insulin receptor, and CD98hc [11]. While these receptors have yielded some success and are transitioning to the clinic with multiple phase I/II clinical trials, there is still room for improvement in both BBB RMT specificity and transport efficiency [12,13,14,15,16,17]. While RNAseq and proteomic studies have improved understanding of BBB gene and protein expression [18,19,20,21], identifying which proteins actually undergo RMT is difficult [22,23]. For that reason, unbiased, phenotypic antibody screens can be a viable and valuable option.

For these unbiased antibody screens to be a valuable tool for the identification and optimization of lead candidates, high-fidelity in vitro model systems are needed. In fact, a major difficulty in the development of antibodies designed to utilize RMT to enter the brain is the potential differential expression of receptor isoforms in murine models when compared to humans [21]. Thus, it is valuable to accurately recapitulate the human RMT expression profile in the in vitro screening substrate. As one option, human-induced pluripotent stem cell-derived brain microvascular endothelial-like cells (hiPSC-derived BMEC-like cells) have proven to be a valuable tool for BBB-targeting antibody screens. BMEC-like cells have been demonstrated to express a diversity of cell surface receptors and transporters characteristic of the human BBB [24,25,26,27], relevant to identifying antibodies recognizing receptors capable of undergoing RMT. For example, they have been used to successfully screen a phage display library of non-immune single-chain variable fragments (scFv) for BBB targeting and transcytosing antibodies [28] and have been used to evaluate transferrin receptor antibody transport [29].

In addition to mammalian IgGs, non-traditional antibody sources have been used in BBB screens to identify novel ligands, including single-chain variable fragments (scFv) [28,30], nanobodies [31,32] sourced from a variety of species from the family *Camelidae* [33], as well as variable lymphocyte receptors [34,35]. Variable lymphocyte receptors (VLRs) serve as the adaptive immune response of the lamprey and hagfish [36,37,38,39]. While the use of VLRs as antibody alternatives is in its nascent stages [40], VLRs have the potential to aid in the identification of novel RMT targets or biomarkers [41], with particular applications in targeting differential glycosylation patterns [42,43]. VLRs have a unique structure when compared to the traditional antibody alternates, with a repeating leucine-rich repeat (LRR) structure commonly seen in toll-like receptors [40,44,45,46]. Monomeric VLRs have a concave horseshoe-like structure with a binding interface whose structure is influenced by the number of LRR variable (LRRV) regions present in the VLR, which can range widely from 0 to more than 20 [40,45,47] (Figure S2). Previous work has used an immunized VLR library against the murine BBB to identify brain vasculature [34] and extracellular matrix-targeting VLRs [35]. Here, we extended the VLR screening platform by creating a VLR library from lamprey immunized with human-induced pluripotent stem cell (hiPSC) derived brain microvascular endothelial (BMEC)-like cells to identify novel VLRs that can target the human BBB and are capable of delivery across the BBB of mice.

## 2. Materials and Methods

### 2.1. Cell Culture

hiPSC-derived BMEC-like cells were cultured and differentiated from IMR90-4 hiPSC(WiCell) [48], as previously described [24,26]. Briefly, IMR90-4 were cultured in TeSR E8 and passaged with Versene and Y-27632, cells were differentiated in unconditioned medium without FGF-2 for 6 days, and cells were transferred into human endothelial serum-free medium supplemented with FGF-2, retinoic acid, and B27 [49]. iPSC-BMEC-like cells were selectively passaged onto collagen IV and fibronectin-coated surfaces. Cells were then utilized 2 days following culture for either live-cell-based assays or fixed with 3.6% paraformaldehyde for experiments utilizing fixed cells, as further described. iPSC-derived BMEC-like cells possess key properties as previously reported [24,26] (Supplemental Figure S1).

hCMEC/D3 (Millipore Sigma, Burlington, MA, USA) cells were cultured according to manufacturer’s protocols. Cells were grown on collagen I-coated flasks in EndoGRO^TM^-MV complete media supplemented with 1 ng/mL FGF-2. Cells were used between passages 4 and 8.

### 2.2. Lamprey Immunization

Three sea lamprey larvae were immunized with fixed iPSC-BMEC-like cells [24] according to previously published methods [34,50]. All lamprey experiments were approved by Emory IACUC and performed at Emory University. Three lamprey were used for this study because previous studies have demonstrated that this number will result in a sufficiently large and diverse pool of VLRs [34]. Lamprey serum was also collected from a non-immunized lamprey as a control. RNA for VLRB was isolated similar to previously described [34]. Briefly, leukocytes and plasma were separated from the blood using a Percoll gradient. RNA was extracted from the leukocytes using the Qiagen RNeasy kit (Qiagen, Germantown, MD, USA). The RNA was converted into cDNA with SuperScript III reverse transcriptase (Invitrogen, Waltham, MA, USA) and oligo-dT priming. VLRB sequences were amplified from leukocyte cDNA by nested PCR using GoTAq^®^ G2 Hot Start DNA polymerase (Promega, Madison, WI, USA). Two rounds of PCR were performed to amplify the VLRB sequences; the first set of PCR had primers covering the N-terminal (CTCCGCTACTCGGCCTGCA) and C-terminal (CCGCCATCCCCGACCTTTG) untranslated regions. The second round of PCR modified the sequence to leave behind the VLRB monomer; the primers were in the LRRNT (GCATGTCCCTCGCAGTG) and LRRCT (CGTGGTCGTAGCAACGTAG).

### 2.3. VLR Library Cloning

VLRBs were cloned into pCT-ESO-BDNF and pCT-ESO-PAS yeast surface display (YSD) vectors as previously described [34,35,51]. Briefly, pCT vectors and VLRB PCR products were digested with NheI, BamHI, and NcoI. EBY100 was inoculated into SD-CAA media with tryptophan and grown overnight at 30 °C. Then, the yeast was grown in YPD from to 1.4 OD_600_ before being washed with cold water and 1 M sorbitol/1 mM CaCl_2_. The yeast was then resuspended in pre-warmed 0.1 M LiAc/10 mM DTT and grown for 30 min at 30 °C, washed twice with cold 1 M sorbitol/1 mM CaCl_2_, and resuspended in 1 M sorbitol/1 mM CaCl_2_. Finally, 9 μg of VLRB PCR product, PAS linker [52], and 3 μg of digested vector (approximately 30:1 insert to vector ratio) was mixed with 400 μL of yeast cells. One hundred microliters of the yeast/DNA mixture was added to a 0.2 cm electroporation cuvette on ice. The yeast was electroporated at 2.5 kV using a BioRad Gene Pulser (Bio-Rad, Hercules CA, USA).

Immediately after electroporation, the yeast was added to a pre-warmed 1:1 mix of YPD and 1 M Sorbitol and grown for 1 h at 30 °C. Then the medium of the yeast was switched to SD-CAA. Four electroporated samples were combined for a library of 9 × 10^7^ clones for the original linker and 6 × 10^7^ clones for the longer PAS linker. Library size was determined by streaking serially diluted libraries on SD-CAA agar plates. Full-length expression of the VLR library was confirmed by flow cytometry epitope labeling of the carboxy-terminal c-*myc* tag.

### 2.4. YSD Library Screening–Biopanning

Yeast was induced and biopanned over the surface of iPSC-BMEC-like cells as previously described [34,53]. Included in parallel with the library were negative controls D1.3 [54] and positive control scFv A [55], which were grown and induced as described above before each biopanning round. Two 6-well plates of iPSC-BMEC-like cells were differentiated and used on day 10 or 11 of the differentiation process. Induced yeast was incubated with the iPSC-BMEC-like cells for 2 h at 4 °C with gentle rocking. Plates were washed with ice-cold PBS–0.1% bovine serum albumin (BSA) 4 times; the D1.3 well was inspected in between each wash to determine if sufficient washing had occurred. Sufficient washing had been achieved when there was no binding in the D1.3 negative control well, but binding remained in the scFv A well. The binding yeast was collected by scraping the cells and placing the collected yeast and iPSC-BMEC-like cells into SD-CAA media to expand the yeast for a subsequent round of biopanning. The yeast was diluted and plated to count yeast recovery for each round. This was repeated three times.

Following the third round of biopanning, yeast was plated on SD-CAA plates and individual yeast clones were isolated; 96 total clones, 48 from the normal linker and 48 from the PAS longer linker libraries. Individual clones were then panned in a 24-well plate using the same protocol was followed for the bulk panning. This was repeated three times. The approximate number of adhered yeast per field of view was measured using bright field microscopy analyzed using the ImageJ particle counting tool (version 2.16.0/1.54p). The number of yeast bound in the negative control condition was subtracted from experimental counts. Data were analyzed using a one-way ANOVA with Dunnett’s multiple comparison test.

### 2.5. Immunolabeling of Tissue and Cells with VLRs

iPSC-BMEC-like cells were incubated in blocking buffer (PBS, 10% goat serum, 1% BSA) (PBSGA) for 15 min. Then the iPSC-BMEC-like cells were incubated with Lamprey Serum (1:10) for 1 h at room temperature. The cells were washed thrice with wash buffer (PBSCM with 1%BSA). The cells were then incubated in Mouse-αVLR (1:100), 4C4, at 4 °C for 1 h. Cells were washed thrice with wash buffer, then incubated in goat-αMouse 488 (1:500) for 30 min at 4 °C. The cells were washed thrice with wash buffer, then post-fixed with 4% PFA. The cells were imaged with a fluorescence microscope (Nikon, Melville, NY, USA).

Brain tissue sections (human and mouse) on slides were washed with PBS, then incubated with blocking buffer (PBS, 1% goat serum, 0.1% BSA, 0.05% saponin) for 30 min. The tissue was incubated with lamprey serum for 1 h at room temperature. Then, the tissue was washed with wash buffer (PBS with 0.05% Saponin). Next, the tissue was incubated with Mouse-αVLR (1:100), 4C4, at 4 °C for 1 h. After the tissue was washed with wash buffer, the tissue was incubated with the secondary antibodies for 30 min on ice goat-αMouse 555 (1:500) and Isolectin B4-Alexa488 (1:100) for mouse tissue, or goat-αMouse 555 (1:500) and CD31 (1:200) for human tissue. The tissue was washed with wash buffer, then post-fixed with 4% PFA. An incubation with DAPI plus 0.05% saponin was conducted to stain cell nuclei. The tissue was mounted on a glass slide with a cover slip and Prolong Gold antifade reagent (Invitrogen), then imaged with a fluorescence microscope.

For soluble VLR-hFc binding, human and mouse brain tissue sections were blocked as described above. A total of 10 µg/mL VLR-hFc was incubated in PBSGA for 1 h at room temperature and washed 3× with wash buffer. The tissue was incubated with the secondary antibodies for 30 min on ice goat-αHuman 555 (1:500) and Isolectin B4-Alexa488 (1:100) for mouse tissue or CD31 (1:200) for human tissue. Quantification was conducted blinded using ImageJ. Regions of interest containing lectin (mouse tissues) or CD31 (human tissues) positive blood vessels were first selected, and then the fluorescence intensity of the VLR-hFc associated with the blood vessels was quantified. Analysis was performed on five fields of view containing a minimum of four vascular segments per VLR. One-way ANOVA with Dunnett’s multiple comparison test was used to compare the VLR-hFc signal to the negative controls.

Human brain sections were obtained in compliance and under the supervision of the University of Wisconsin-Madison Institutional Review Board (IRB). De-identified normal human brain tissue was obtained from individuals undergoing surgery for other indications. Patients consented to the surgery at the University of Wisconsin Hospital, including consent for the research use of tissue removed during surgery.

### 2.6. Fc-Fusion Protein Production

Candidate VLRs identified in the screen were cloned into the pFUSE-hIgG1-Fc2 vector originally containing DO11.10 β-(G_4_S)_4_-α scFv. VLRs were amplified using primers AGAGAGAATTCGTGTCCCTCGCAGTGTTC and AGAGAACCGGTCGTGGTCGTAGCAACGT, the pFUSE backbone and VLR PCR products were digested with EcoRI and AgeI before being ligated using T4 ligase and transforming dh5-alpha Mix go cells, which were plated on LB agar plates containing zeocin. Candidate VLRs were also cloned into the pIRES vector containing rabbit Fc-LL-neurotensin [30].

VLR-Fc and VLR-Fc-NT fusion proteins were produced in ExpiCHO cells following the manufacturer’s recommended protocol and were grown for a period of 5–7 days before the protein was harvested. Protein was purified using agarose a/g beads, as previously described [30,34]. Purified protein products were run on SDS-page gels and stained to validate that the appropriate-sized protein was produced. Neurotensin fusion proteins were tested for activity using the DiscoverX NT1 Human Neurotensin GPCR Cell-Based Agonist kit (Eurofins Discovery, Fremont, CA, USA) according to manufacturer specifications [30].

### 2.7. Cell-Based Assays

Binding of the VLR-Fc proteins was carried out in hCMEC/D3 [56] (Millipore Sigma SCC066). In all cell-based assays, cells were serum starved in complete media lacking serum for one hour, then exposed to 10 µg/mL VLR-Fc protein for 30 min. Following the incubation period, cells were washed 3× with ice-cold PBS and incubated with goat-anti-human IgG-Alexa488 in PBSGA for 30 min on ice. Cells were then washed 3× in ice-cold PBS and fixed in 4% PFA for 10 min at room temperature. Cells were then washed 3× with PBS on ice before nuclei were labeled with DAPI. Cells were then imaged on a Nikon microscope. Experiments were completed with three separate passages of cells with five fields of view per VLR-hFc. Whole image intensity was quantified using ImageJ, and a one-way ANOVA with Dunnett’s multiple comparison test was used to compare the VLR-hFc signal to the negative controls.

### 2.8. Animal Experiments

All experiments performed with mice were in accordance with the University of Wisconsin IACUC-approved animal use protocol. For lead candidate evaluation, candidate VLRs were injected intravenously via tail vein injection at a concentration of 10 mg/kg and allowed to circulate for one hour in two C57BL/6 male mice, approximately 18–20 g. Mice were stored in standard housing, including free access to food and water, with environmental enrichment materials. Following circulation, mice were anesthetized with ketamine xylazine and transcardially perfused with a saline solution containing heparin and lectin, as described previously [34], and subsequently by the same perfusate containing 4% paraformaldehyde. Organs were then removed and flash frozen. All tissues were then sectioned on a Leica CM1950 cryostat in the TSB-Biobank at the University of Wisconsin, and tissues were sectioned at a thickness of 30 µm for lung and 8 µm for all other tissues. Tissue sections were then stained similarly as described above; tissue was blocked in blocking buffer for 1 h, then incubated overnight at 4 °C with goat-anti-human IgG-Alexa568, then washed three times with PBS before mounting with Prolong Gold DAPI and imaging. Vascular labeling was measured blinded using ImageJ. Blood vessels were first identified using lectin as a vascular marker, and the fluorescence intensity of VLR-hFc associated with the blood vessels was measured. A minimum of five images from two mice were analyzed, totaling between 100 and 200 blood vessels per condition. Biodistribution was quantified by looking at the whole image intensity of five fields of view for each organ using ImageJ. Data was analyzed using a one-way ANOVA with Dunnett’s multiple comparison test.

Neurotensin experiments were performed, as previously described [30]. Briefly, mice had Star-Oddi nanoDST loggers implanted in their intraperitoneal cavity 5 days before injections. Internal temperature readings were taken every 30 min for the 24 h before animals were injected with 10 or 20 mg/kg neurotensin fusion proteins by tail vein injection. Body temperature measurements were taken every 5 min beginning 2–3 h before the injection and continued for 12 h before decreasing in frequency to every 15 min before returning to every 30 min 12 h later. Mice were sacrificed, and the loggers were removed 48 h post-injection. Cage and littermates were used when possible, cages were stored on the same position in the rack, and timing was consistent across all experiments. Animals were observed at least every 24 h for signs of distress; none were observed. No animals were excluded from analysis. Mice were assigned to randomized groups, and the data was analyzed blinded. Between 4 and 7 mice were used per condition. Data was analyzed using a two-way ANOVA with a Bonferroni multiple comparison test.

### 2.9. Imaging

Microscopy was performed on a Nikon TiE microscope, utilizing a 40× objective and NIS Elements software. Images were analyzed in ImageJ.

### 2.10. Statistical Analysis

Statistical significance was tested using a variety of one- or two-way ANOVA tests with appropriate post hoc multiple comparison analysis using BioRender Graph https://www.biorender.com/ (accessed on 5 September 2025) and Microsoft Excel (Version 16.100.3); the specific analysis type has been notated in the figure captions containing the relevant data. The sample size was decided based on previous experience and the expected effect size.

## 3. Results

**Figure 1:**
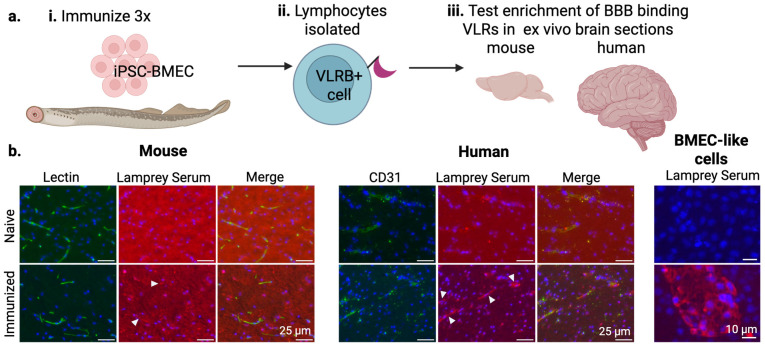
Flowchart of the initial steps of antibody library generation. (**a**) (**i**) Lamprey were immunized with intact iPSC-BMECs, (**ii**) lymphocytes were harvested, and (**iii**) pooled lamprey serum was incubated with mouse and human brain cryosections and iPSC-BMEC-like cells to confirm BBB immunoreactivity. (**b**) Mouse brain sections were labeled with lectin (green) to indicate vasculature and stained with DAPI (blue) to label nuclei. Human brain sections were stained with DAPI (blue) to label nuclei and CD31 (green) to label vasculature. White arrowheads indicate examples of vascular co-localization. BMEC-like cells were fixed and labeled with pooled lamprey serum (red) and DAPI (blue).

To generate VLRs that target the human BBB, larval lamprey were immunized with whole, fixed iPSC-derived BMEC-like cells, as lamprey have been shown to have robust immune responses in response to whole or fragmented cells [34,50]. Six weeks following immunization, the serum and lymphocytes were harvested from three immunized lamprey (Figure 1a). To ensure that the iPSC-derived BMEC-immunized lamprey produced VLRs that are capable of binding the iPSC-derived BMEC-like cells used as immunogens, as well as the in vivo BBB, pooled lamprey sera were incubated with human brain cryosections and cultured iPSC-derived BMEC-like cells (Figure 1b). As expected, VLR-containing sera bound strongly to a subset of the iPSC-derived BMEC-like cells. Importantly, the sera also bound to human brain vasculature when compared to the naïve lamprey serum, indicating in vivo relevance to VLRs in the lamprey immune repertoire. Additionally, we assayed for mouse cross-reactivity as this would be important for in vivo testing and validation of lead candidates. Indeed, the sera also labeled mouse brain vasculature, albeit with a qualitatively weaker signal, which was unsurprising given that human-sourced cells were used for immunization.

**Figure 2:**
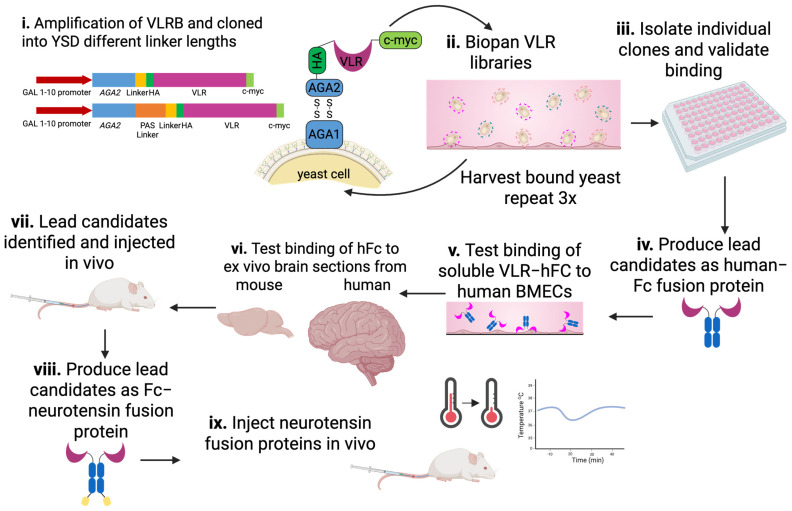
Flowchart of the antibody screen and validation process. (**i**) The VLRB mRNA was amplified from isolated lamprey lymphocytes and inserted into the pCT YSD plasmid. (**ii**) YSD libraries were panned over the surface of iPSC-BMECs three times before (**iii**). Individual YSD clones were identified and validated. (**iv**) VLRs were then cloned into mammalian Fc fusion protein constructs and produced in CHO cells. (**v**) VLR-Fcs were tested for binding to the hCMEC/D3 BBB model and for (**vi**) binding to mouse and human brain cryosections. (**vii**) Lead candidates were injected into mice to identify luminal binding VLRs for further evaluation. To ensure that VLRs are able to sufficiently penetrate into the brain parenchyma, VLRs were (**viii**) cloned as VLR-Fc-neurotensin (NT) fusion proteins. (**ix**) NT fusion proteins were injected into mice with a temperature logger in the intraperitoneal cavity to monitor body temperature. Brain-penetrant VLRs cause transient hypothermia if capable of successfully undergoing receptor-mediated transcytosis at the BBB and delivering NT to the brain.

Given the observable immune response, VLRB-encoding genes were amplified from the isolated lymphocytes pooled from the three immunized lamprey. VLRs were then subcloned into the yeast surface display (YSD) system for screening (see Materials and Methods for details). In this way, two YSD libraries were created, with one having the standard short linker between the Aga2p tether protein and the displayed VLR (16 amino acid G_4_S linker, library size 9 × 10^7^), and a second with an additional 38 amino acid long PAS linker (library size 6 × 10^7^) (Figure 2i). The longer linker adds flexibility and cell surface accessibility to YSD, which could potentially increase the diversity of screened VLRs against their cell surface targets [52].

In order to identify VLRs capable of targeting the human BBB that could also be subject to in vivo validation in a murine model, we screened the lamprey VLR YSD libraries and validated potential hits as outlined in Figure 2. To identify VLRs that specifically bound the iPSC-BMEC-like cell immunogen, yeast were first biopanned against monolayers of iPSC-BMEC-like cells, and cell-associated yeast-displaying VLR clones were recovered in each round and expanded as previously described [34] (Figure 2ii).

**Figure 3:**
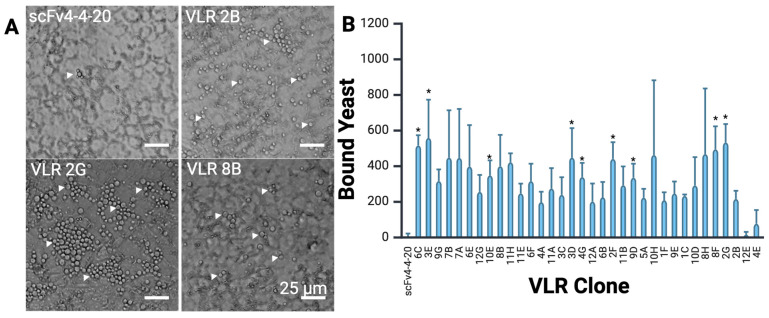
Yeast surface display (YSD) biopanning to analyze individual VLR clones. (**A**) Images from YSD binding of re-transformed clones show a range of yeast binding to iPSC-BMEC-like cells, from minimal binding on the negative control scFv4-4-20, to clear yeast binding for individual VLR-displaying clones. Yeast are the spherical objects on top of the iPSC-BMEC-like cell monolayer, and a few are indicated in each image by white triangles. To facilitate quantification of bound yeast, cells were imaged at the focal plane containing the cross-section of yeast; as a result, out-of-focus iPSC-BMECs are found in the background. (**B**) Quantification of the number of yeast seen on the surface of iPSC-BMECs per field of view, negative control scFv4-4-20 binding has been subtracted from these values to account for background yeast binding, mean ± S.D. Data was analyzed using one-way ANOVA with Dunnett’s multiple comparison test, significance indicated on the graph corresponds to * *p* < 0.05, *n* = 3 microscope fields.

After three rounds of biopanning, individual VLR clones were isolated and sequenced. An initial sample of 96 clones was sequenced, resulting in a total of 34 unique VLR sequences with the variable binding interface varying in length from 1 to 4 LRRVs (Figure S1). Eleven identical VLR clones were identified from both the normal and long linker libraries, suggesting the validity of these clones; and accordingly, sampling of two representative clones displayed with either the normal or long linker indicated that these clones showed no differences in yeast binding to iPSC-BMEC-like cells (Figure S3). In addition, there were VLR clones that were uniquely identified in either the normal or long linker library. Retransformation of individual VLR clones into the parent yeast display strain and biopanning verified that the observed binding was due to the VLR clones themselves, rather than any spurious yeast mutations (Figure 3). While only 9 of 34 clones showed a statistically significant increase in binding compared to the negative control, all 34 clones were moved forward into protein production for the evaluation of soluble VLRs, given the observed yeast binding to iPSC-BMEC-like cells (Figure 2iv).

**Figure 4:**
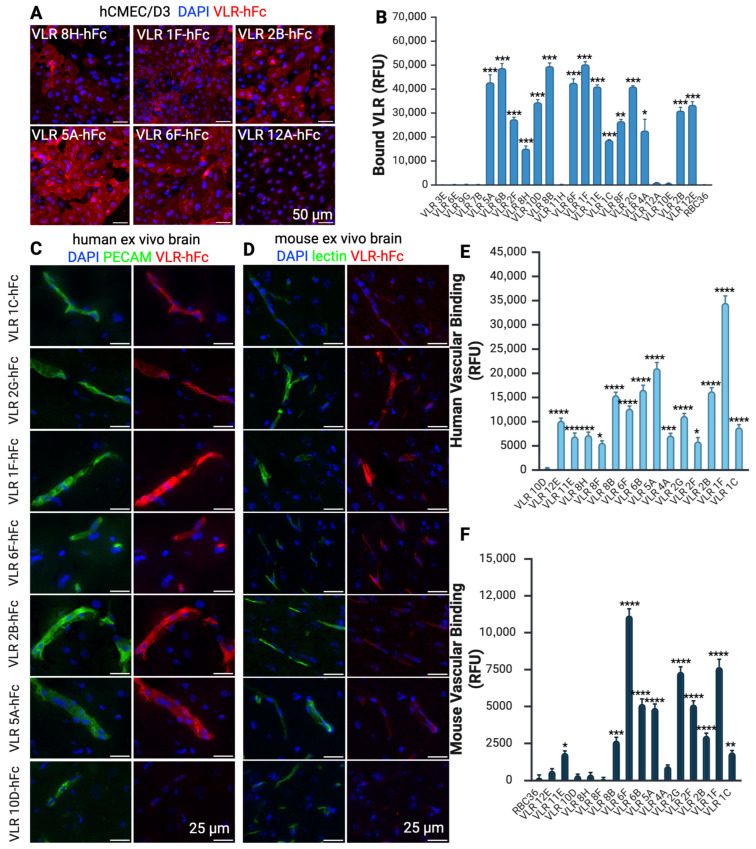
VLR-hFc binding to hCMEC/D3 cells and human and mouse brain cryosections. (**A**) Representative images showing hCMEC/D3 cell surface binding of a subset of VLR-hFcs (red) with nuclei labeled with DAPI (blue). (**B**) Quantitation of binding for all tested VLR-hFcs to hCMEC/D3 cells. All samples were compared to the negative control VLR, RBC36-hFc, using a one-way ANOVA with Dunnett’s multiple comparison test. (**C**) Images of human brain cryosections incubated with 10 µg/mL VLR-hFc (red) to show binding with brain vasculature immunolabeled for CD31 (green) and nuclei (blue). (**D**) Representative images of mouse brain cryosections perfused with vascular binding lectin (green) were incubated with 10 µg/mL VLR-hFc (red) to show binding to mouse brain vasculature. (**E**) Quantification of the vascular labeling of VLR-hFc on human ex vivo brain sections. All samples were compared to VLR10D-hFc using a one-way ANOVA with Dunnett’s multiple comparison test. (**F**) Quantification of the vascular labeling of VLR-hFc on mouse ex vivo brain sections. All samples were compared to VLR RBC36-hFc using a one-way ANOVA with Dunnett’s multiple comparison test. Significance indicated on the graphs corresponds to * *p* < 0.05, ** *p* < 0.01, *** *p* < 0.001, **** *p* < 0.0001. A full property listing for all VLR clones can be found in Table S1.

Of the 34 VLRs identified in YSD, 22 were successfully produced as fusion proteins to the human Fc region (VLR-hFc) in CHO cells (Figure S4). VLR-hFcs were then evaluated for binding to human immortalized BMECs (hCMEC/D3) (Figure 2v). This BBB model was used to ensure that any VLR-hFc was not simply binding to an iPSC BMEC-like cell target that could be an artifact of the iPSC differentiation process. More than two-thirds (15/22) of the VLR-hFc constructs produced strong binding to the surface of hCMEC/D3 cells, whereas some VLR-hFc (e.g., 12A and 6E) did not bind and were not explored further (Figure 4A,C, Table S1).

To confirm that the VLR clones bind to target antigens that are expressed at the BBB in vivo, the 15 positive hCMEC/D3 binders were incubated with human and mouse brain cryosections. Number 14/15 showed clear vascular binding to human brain tissue (Figure 4C, Table S1), while 10/15 also exhibited binding to mouse tissue (Figure 4D, Table S1). Five VLRs were selected for further analysis that showed strong binding to human and mouse tissue (VLR 1F, 2B, 6F, 2G, and 5A).

**Figure 5:**
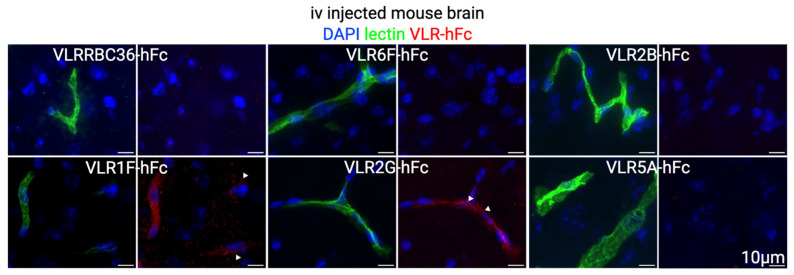
Immunofluorescence images showing VLR binding (red) colocalized with brain vasculature (green) and nuclei (blue). Lead candidates were intravenously injected in mice at a concentration of 10 mg/kg (red) and allowed to circulate for one hour before mice were sacrificed and perfused with lectin (green) for vascular labeling. White arrowheads indicate regions of visible punctate staining.

**Figure 6:**
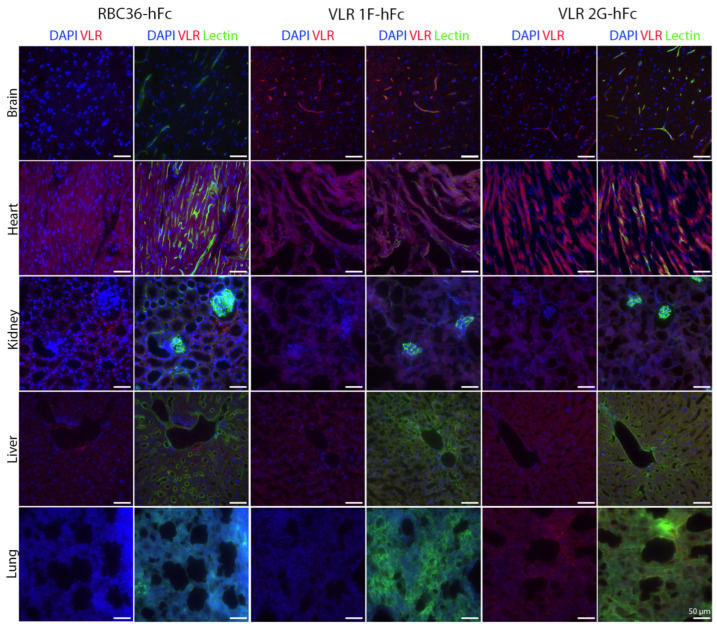
VLR-hFc constructs were intravenously injected into mice at a concentration of 10 mg/kg, and the antibody was allowed to circulate for one hour. Mice were then sacrificed and perfused with lectin (green) to label the vasculature, the mice were dissected, and the tissues were snap frozen, sectioned, and immunolabeled for VLR-hFc (red) and nuclei (DAPI, blue).

The five selected VLR clones were next tested for their capacity to target luminal BBB antigens after intravenous injection. To this end, VLR-hFcs were intravenously injected into mice at 10 mg/kg and allowed to circulate for one hour. Immunofluorescence assessment of the injected brains verified that 2 VLR-hFcs could localize to the BBB (1F and 2G, Figure 5 and Figure S5). Both of these VLRs showed punctate staining within the vasculature, suggesting possible internalization and BBB trafficking. Preliminary biodistribution analyses suggest that VLR2G-hFc is not brain specific, with increased binding within the lung, although vascular localization was only observed in the brain (Figure 6 and Figure S6). By contrast, despite not having any negative selection for VLRs that bind to other vascular beds, VLR1F-hFc does appear to be brain selective, with similar uptake in other organs as negative control RBC36-hFc. We also observed increased intensity in VLR binding to non-vascular structures in the heart for all VLRs, including the negative control RBC36-hFc.

**Figure 7:**
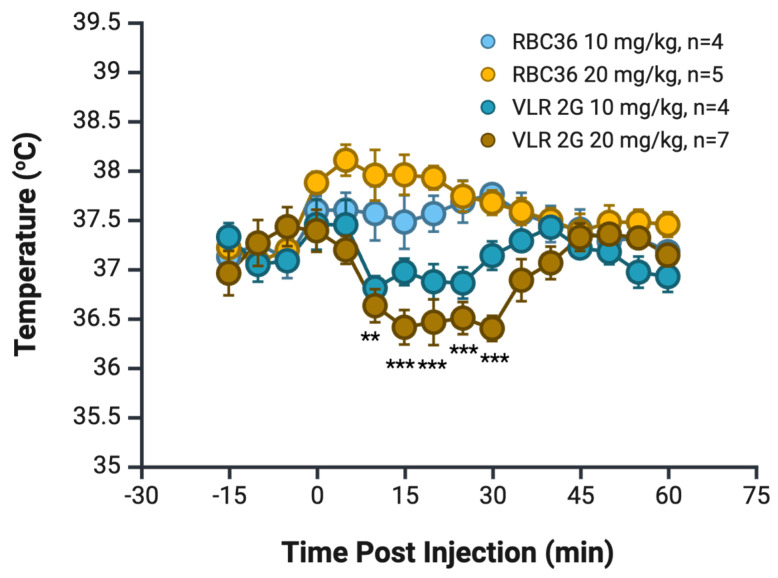
Hypothermia was observed in mice following iv injection of VLR 2G-rbFc-NT in a dose-dependent manner. Temperature was recorded every 5 min, and VLR 2G-rbFc-NT 20 mg/kg showed a significant decrease in temperature compared to both doses of RBC36-rbFc-NT negative controls. Two-way ANOVA was used with Bonferroni multiple comparison test, significance indicated on the graph corresponds to ** *p* < 0.01, *** *p* < 0.001 between VLR2G 20 mg/kg and both RBC36 control groups. Minimum of *n* = 4 mice.

Finally, to test whether the VLRs fully transcytosed into the brain and if they could do so at levels sufficient to elicit a pharmacologic response, we used the previously described neurotensin-based hypothermia mouse model that has been used to validate brain parenchymal uptake [30,57,58]. When NT is administered peripherally, its effects in the CNS are limited, but if an antibody facilitates its entry into the CNS at sufficient concentrations, transient hypothermia is observed [30,57]. For these experiments, VLRs 1F and 2G were fused to a rabbit Fc region that is linked at the carboxy-terminus with neurotensin, a construct previously validated by our laboratory [30]. Mice were tail vein injected at a dose of 10 mg/kg with RBC36-rbFc-NT negative control or the lead candidates VLR1F-rbFc-NT or VLR2G-rbFc-NT. Mice injected with VLR2G-rbFc-NT exhibited a maximum temperature drop of 0.8 °C compared to dose-matched controls at 25 min post-injection (*p* = 0.012), subsequently returning to the baseline of the RBC36-rbFc-NT negative control (Figure 7). When the dose was increased to 20 mg/kg, a deepened decrease in temperature was observed with a 1.6 °C drop in temperature when compared with a dose-matched RBC36-rbFc-NT negative control (*p* = 0.00004), again at approximately 25 min post-injection (Figure 7). In contrast to the situation observed with VLR2G-rbFc-NT, mice injected with VLR1F-rbFc-NT did not exhibit any signs of transient hypothermia and instead maintained an elevated body temperature (1 °C above a dose-matched negative control) for six hours post-injection (Figure S7), although no other visible signs of distress were seen in the animals. Taken together, VLR clone 2G exhibits the most promise as a BBB delivery vehicle given that is binds to a target expressed at both the in vivo human and mouse BBB and can deliver a pharmacologic payload.

## 4. Discussion

In this work, libraries of VLRs generated through immunization of lamprey with iPSC-derived BMEC-like cells were screened using YSD biopanning on the surface of the BMEC-like cells. Using a combination of BBB models and human and murine brain sections, selected VLR clones displayed in vivo-relevant BBB binding and species cross-reactivity, allowing for further testing in mice while preserving the capability for potential future translation. Given our filtering criteria, some number of VLR clones that were excluded due to poor cross-reactivity to the mouse BBB may still be of interest for human BBB targeting.

An iPSC-derived model of BMEC-like cells was employed as a BBB model, and this resulted in an improved percentage of VLRs capable of human BBB binding in tissue sections (93%) compared to our previous study based on lamprey immunization with murine brain microvessel fragments (62% [34]). In fact, these results are similar to those achieved with a human scFv library screened against these same BMEC-like cells (83% [28]). While these BMEC-like cells have elevated epithelial characteristics and decreased endothelial phenotype compared to physiological BMECs [59], they have proven to be a reasonable model of BBB transport [24,26,60]. Here, we have reaffirmed their relevance and utility in a screen for VLRs that have human in vivo BBB binding capability. In addition, this screen did not contain a filter to reduce non-brain-specific targeting, and accordingly, VLR2G showed increased binding in the lung, yet VLR1F showed limited binding in other vascular beds. VLR2G appears most promising for brain drug delivery, as demonstrated by the VLR2G-rbFc-NT conjugate. Further filtering of the initial VLR libraries could include a subtraction step for binding to non-brain endothelial cells to enhance BBB specificity [61].

The use of neurotensin in a pharmacodynamic setting to validate parenchymal penetration of VLR 2G confirms the potential use of this VLR as a drug delivery carrier. On its own, neurotensin has limited ability to cross the BBB and enter the brain, but upon direct injection into brain tissue, it can have hypothermic or antinociceptive effects depending on the brain region [62]. As such, neurotensin has been used as a model cargo for assessing BBB penetration. Here, we show the advantage of using neurotensin conjugation assays in the screening of BBB-targeting proteins with two very different VLRs, which likely bind to different targets, given their different peripheral organ binding profiles, with VLR1F showing more brain specificity. VLR1F also displays qualitatively higher brain vascular binding in comparison to VLR2G. However, when these two VLRs were tested using the neurotensin pharmacodynamic assay, only VLR2G produced the anticipated hypothermia response for a BBB crossing targeting molecule, whereas VLR1F produced hyperthermia. The precise cause of this hyperthermia is unknown. The neurotensin assay has also been used to test brain uptake of optimized Angiopep-2-based peptides [58], transferrin-targeting nanobodies [57,63,64], and low-density lipoprotein receptor (LDLR)-targeting cyclic peptides [65]. Specifically, when looking at transferrin-targeting nanobodies, it was shown that this method was able to select between engineered nanobody anti-transferrin nanobody-NT fusions, improving the hypothermic response from a modest 0.5 °C hypothermic response for the wild-type nanobody to a 6 °C drop in body temperature that lasted for nearly five hours before the optimal engineered variant [57]. Similarly, an angiopep-2 peptide engineered for optimal analgesic effect was shown to produce a 6 °C drop in body temperature for slightly over 5 h [58]. By comparison, the relatively modest decrease in body temperature elicited by the 2G-rbFc-NT fusion emphasizes that this lead VLR was selected for BBB binding and has not yet been optimized for BBB transport. As has been shown with transferrin-targeting antibodies, engineering for affinity optimization or pH binding sensitivity can dramatically improve brain penetration and uptake [63,66]. While we have not explored the detailed binding properties of the VLRs in this lead identification campaign, it is possible that similar engineering strategies could be used to further optimize the brain drug delivery properties of VLR2G.

Finally, for VLR2G to be further advanced as a BBB delivery agent, issues of potential immunogenicity and target identification would need to be addressed. While identification of the binding partners for VLR2G was outside of the scope of the current work, it will be an important next step. Therefore, we cannot rule out that VLR2G targets one of the commonly targeted BBB receptors, such as CD98hc, insulin receptor, or transferrin receptor. It is also possible that these VLRs may bind to glycoepitopes as previously demonstrated with VLRs from a similar screen [34]. To identify the target of VLR2G, potential approaches could include immunoprecipitation coupled with mass spectroscopy [67], competition assays with ectodomains of known BBB targets, or binding to glycoarrays [34]. Irrespective, VLR2G is a new targeting molecule that could display differences in targeting, CNS uptake, and biodistribution. Thus, while VLR2G is a promising new brain delivery agent, future work will be needed for eventual translation.

## Data Availability

The original contributions presented in this study are included in the article/Supplementary Materials. Further inquiries can be directed to the corresponding author.

## Supplementary Materials

The following supporting information can be downloaded at https://www.mdpi.com/article/10.3390/pharmaceutics17091179/s1, Table S1. Summary describing the properties of lead VLR-Fc candidates. + indicates positive outcome, − indicates negative outcome, blank boxes indicate the experiment was not conducted. Figure S1. iPSC BMEC-like cell characterization. iPSC BMEC-like cells express key markers as previously reported [24]. Figure S2. Ribbon structure of VLR P1C10 (PDB 9CJ0) [45] showing the modular LRR structure of a standard VLR. Starting with LRRNT (red), LRR1 (orange), LRRV1 (yellow), LRRVe (green), CP (teal), to LRRCT (blue). The LRRV unit (yellow) in the center can be expanded to contain additional LRRV modules; the number of these repeats found in the VLRs investigated here is summarized in the table. Underneath is the amino acid sequence of the two lead VLRs investigated in this work. Figure S3. Two representative yeast-displayed VLR clones, which were identified in both the normal (NL) and longer linker (LL) length libraries, were biopanned against hBMEC-like cells, and bound yeast was quantified, mean ± S.D., *n* = 3 fields, 2-way ANOVA with Bonferroni multiple comparisons test. Figure S4. Reduced SDS-page, Coomassie-stained gel of purified VLR-hFc proteins showing production of VLR-hFcs for a sampling of VLR-hFc constructs. Arrow indicates the 45–60 kDa size range expected for each arm of the various VLR-hFc fusions. Figure S5. Quantification of vascular intensity is presented in Figure 5 for i.v.-injected VLRs demonstrating that only VLR 1F and 2G maintain BBB binding. All samples were compared to negative control VLR, RBC36-hFc using a one-way ANOVA with Dunnett’s multiple comparison test, with significance indicated on the graph corresponding to **** *p* < 0.0001. Figure S6. Quantification of data is presented in Figure 6, whole image intensity for VLR1F and 2G compared to the negative control VLR RBC36. One-way ANOVA was used with Dunnett’s multiple comparison test; significance indicated on the graph corresponds to * *p* < 0.05. Figure S7. Mouse temperature following VLR1F-rbFc-NT injection at 10 mg/kg showing sustained elevated temperature compared to RBC36-rbFc-NT negative control, *n* = 4.

## Abbreviations

The following abbreviations are used in this manuscript:

BBB	Blood–brain barrier
hiPSC	Human-induced pluripotent stem cells
BMEC	Brain microvascular endothelial cell
VLR	Variable lymphocyte receptor
RMT	Receptor mediate transcytosis
RNAseq	Ribonucleic acid sequencing
scFv	Single chain variable fragments
IgG	Immunoglobulin G
LRR	Leucine-rich repeat
LRRV	Leucine-rich repeat variable
FGF-2	Fibroblast growth factor 2
YSD	Yeast surface display
BSA	Bovine serum albumin
PBSGA	Phosphate-buffered solution with goat serum and bovine serum albumin
IRB	Institutional review board
LL	Longer linker
CHO	Chinese hamster ovary cells
NT	Neurotensin
SDS	Sodium dodecyl sulfate polyacrylamide gel electrophoresis
Fc	Fragment crystallizable
PFA	Paraformaldehyde
IACUC	Institutional animal care and use committee
hFC	Human Fc
rbFc	Rabbit Fc
CNS	Central nervous system
LDLR	Low-density lipoprotein receptor
