# Supplementary material for: Identification of Variable Lymphocyte Receptors That Target the Human Blood–Brain Barrier
Source: Pharmaceutics. 2025 Sep 10;17(9):1179. doi: 10.3390/pharmaceutics17091179 (PMC12473985; doi:10.3390/pharmaceutics17091179)
Supplement: Supplementary file 1 [file pharmaceutics-17-01179-s001.zip › pharmaceutics-3781011-supplementary.pdf]

pharmaceutics

Identification of Variable Lymphocyte Receptors that Target the Human Blood-Brain Barrier

Katt, Moriah E.[1,2,3*], Waters, Elizabeth A.[1], Gastfriend Benjamin D.[1], Herrin, Brantley R. [4], Cooper, Max D.[4], Shusta, Eric V. [1,5]

 https://doi.org/10.3390/pharmaceutics17091179

Table S1. Summary describing the properties of lead VLR-Fc candidates. + indicates positive outcome, - indicates negative outcome, blank boxes indicate the experiment was not conducted.

Candidate VLRs	hCMEC/D3 binding	ex vivo human brain binding	ex vivo mouse brain binding	iv mouse brain binding	NT hypothermia
3E	-				
6E	-				
9G	-				
7B	-				
5A	+	+	+	-	
6B	+	+	+		
2F	+	+	+		
8H	+	+	-		
10D	+	-	-		
8B	+	+	+		
11H	-				
6F	+	+	+	-	
1F	+	+	+	+	-
11E	+	+	+		
1C	+	+	+		
8F	+	+	-		
2G	+	+	+	+	+
4A	+	+	-		
12A	-				
10E	-				
2B	+	+	+	-	
12E	+	+	-		

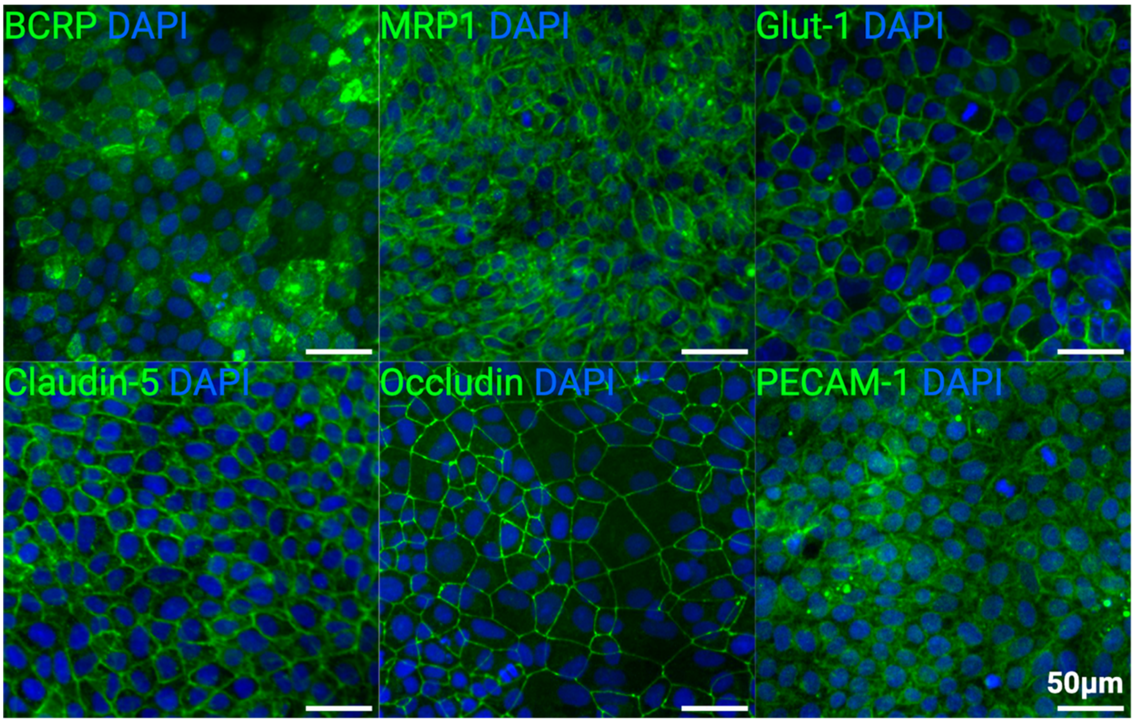

Figure S1. iPSC BMEC-like cell characterization. iPSC BMEC-like cells express key markers as previously reported [24].

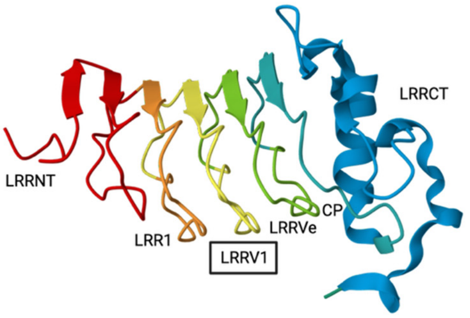

	Normal Linker	Longer Linker	Total (Unique)
1LRRV	1	0	1
2 LRRV	5	2	6
3 LRRV	17	16	24
4 LRRV	3	1	3
Total	26	19	34

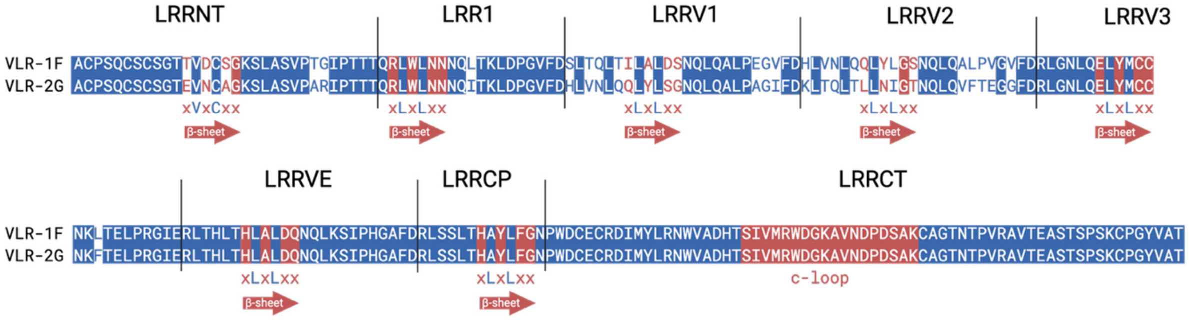

Figure S2. Ribbon structure of VLR P1C10 (PDB 9CJ0)[45] showing the modular LRR structure of a standard VLR. Starting with LRRNT (red), LRR1 (orange), LRRV1 (yellow), LRRVe (green), CP (teal), to LRRCT (blue). The LRRV unit (yellow) in the center can expanded to contain additional LRRV modules, the number of these repeats found in the VLRs investigated here are summarized in the table. Underneath is the amino acid sequence of the two lead VLRs investigated in this work.

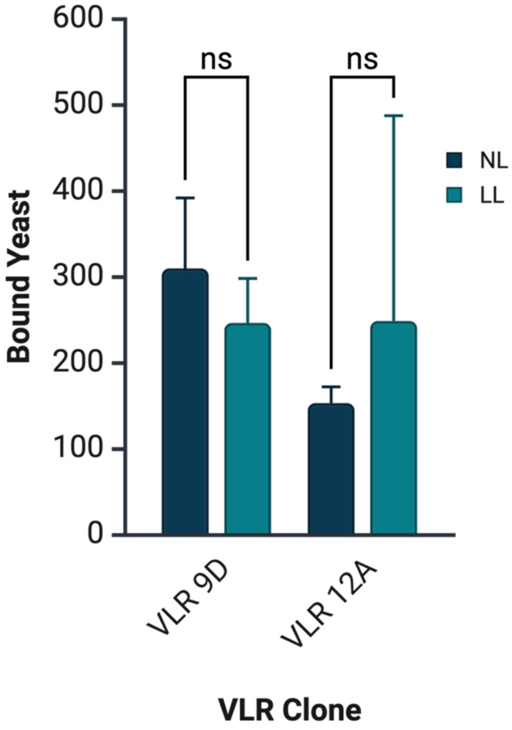

Figure S3. Two representative yeast-displayed VLR clones which were identified in both the normal (NL) and longer linker (LL) length libraries were biopanned against hBMEC-like cells and bound yeast quantified, mean±S.D., $n = 3$ fields, 2-way ANOVA with Bonferroni multiple comparisons test.

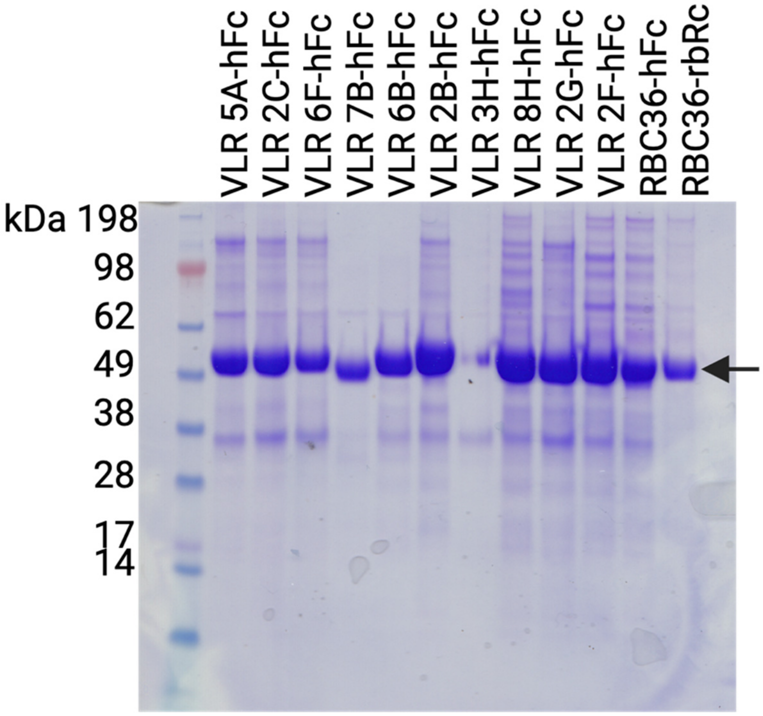

Figure S4. Reduced SDS-page, Coomassie-stained gel of purified VLR-hFc proteins showing production of VLR-hFcs for a sampling of VLR-hFc constructs. Arrow indicates the 45-60 kDa size range expected for each arm of the various VLR-hFc fusions.

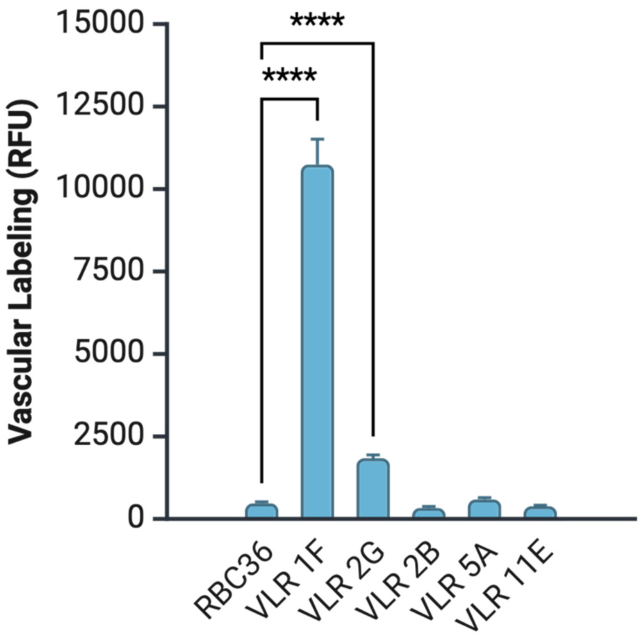

Figure S5. Quantification of vascular intensity presented in Figure 5 for i.v. injected VLRs demonstrating that only VLR 1F and 2G maintain BBB binding. All samples were compared to negative control VLR, RBC36-hFc using a One-way ANOVA with Dunnett's multiple comparison test, with significance indicated on the graph corresponds to ****$p < 0.0001$.

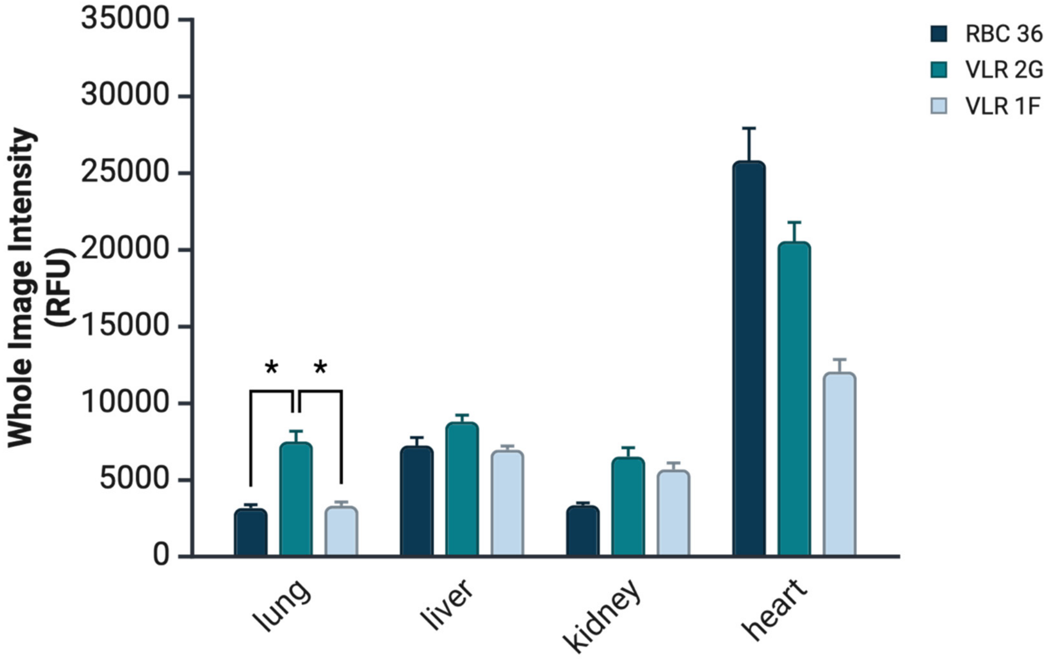

Figure S6. Quantification of data presented in Figure 6, whole image intensity for VLR1F and 2G compared to the negative control VLR RBC36. One-way ANOVA was used with Dunnett's multiple comparison test, significance indicated on the graph corresponds to * $p < 0.05$.

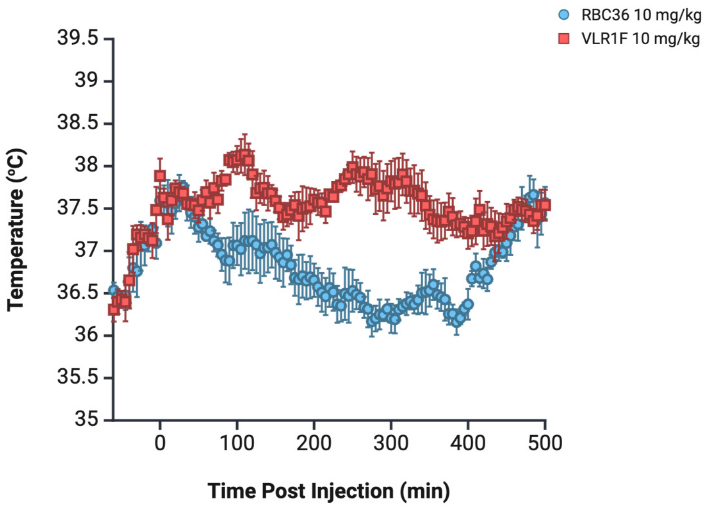

Figure S7. Mouse temperature following VLR1F-rbFc-NT injection at 10 mg/kg showing sustained elevated temperature compared to RBC36-rbFc-NT negative control, *n* = 4.
